# A meta‐analysis investigating the efficacy and adverse events linked to sacubitril‐valsartan in various heart failure subtypes

**DOI:** 10.1002/clc.24192

**Published:** 2023-11-27

**Authors:** Qing Ji

**Affiliations:** ^1^ Nursing College Zibo Vocational Institute Zibo Shandong China

**Keywords:** ACE Inhibitors, heart failure, HFpEF, HFrEF, sacubitril‐valsartan

## Abstract

**Background:**

Sacubitril‐valsartan, an inhibitor of the angiotensin receptor neprilysin (ARNi), has been purported to exhibit superiority over angiotensin converting enzyme (ACE) inhibitors and angiotensin receptor blockers (ARBs) in individuals diagnosed with heart failure.

**Hypothesis:**

This paper gives an updated meta‐analysis comparing the efficacy and safety of sacubitril‐valsartan to that of standard treatment for different types of heart failure.

**Results:**

The meta‐analysis comprised a total of nine randomized controlled trials (RCTs), incorporating data from a substantial sample size of 15 939 patients. The study observed a decrease in overall mortality and mortality related to cardiovascular causes among patients in the heart failure with reduced ejection fraction (HFrEF) category who were treated with sacubitril‐valsartan. However, no statistically significant variation in this outcome was seen among patients with heart failure with preserved ejection fraction and HFmrEF. Patients who were administered sacubitril‐valsartan had a notably elevated likelihood of experiencing hypotension. Nevertheless, no significant disparities were observed in terms of other adverse events among the various treatment groups.

**Conclusion:**

Current meta‐analysis provide support for use of sacubitril‐valsartan in decreasing mortality in patients with HFrEF. However, more numbers of studies are required to draw a definite conclusion on other benefits associated with sacubitril‐valsartan use over standard treatment of ACE inhibitors and ARBs.

## Graphical abstract

Heart failure with reduced ejection fraction [HFrEF] has left ventricular dilatation and unfavorable cardiac remodeling with LVEF < 40%. Sacubitril‐valsartan is the first‐line treatment for HFrEF by American College of Cardiology (ACC)/American Heart Association (AHA)/Heart Failure Society of America (HFSA).

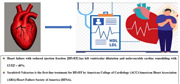

## 1 INTRODUCTION

The American Heart Association and the American College of Cardiology describe heart failure (HF) as “a complicated clinical illness that can emerge from any anatomical or functional cardiac problem that limits the ventricle's capacity to fill with or evacuate blood.”^1^, ^2^ Dyspnea, tiredness, and indicators of volume overload, such as peripheral edema, are all symptoms. Heart failure (HF) can be attributed to a multitude of reasons, encompassing systemic illnesses, diverse cardiac abnormalities, and certain hereditary conditions.^3^

Globally, an estimated 64.3 million people have HF, with numbers expected to climb as the population ages and new therapies for hypertension, diabetes, and other conditions become accessible.^2^, ^4^, ^5^ It is projected that HF will have an impact on a population of around 8 million individuals in the United States by the year 2030, which would represent approximately 3% of the total population.^4^ The incidence of HF is significantly higher in the elderly population, as evidenced by the fact that individuals aged 65 and above make up 80% of hospitalizations related to HF and 90% of deaths associated with HF.^6^, ^7^

The rising frequency of HF places a considerable financial strain on healthcare systems, as it is linked to both direct and indirect medical expenditures. Based on a study conducted in 2012, it was determined that the annual economic cost of HF amounted to $108 billion.^8^ According to a comprehensive study conducted in 2020, the yearly median overall medical expenses for HF patients were $24 383 per patient, including HF‐specific hospitalization costs ($15 879 per patient). Furthermore, individuals with HF with reduced ejection fraction (HFrEF) had greater expenditures than those with heart failure with preserved ejection fraction (HFpEF).^9^

HF is categorized into different subtypes according to the left ventricular ejection fraction (LVEF). These subtypes include heart failure with reduced ejection fraction (HFrEF) where the LVEF LVEF ≤ 40%, HFpEF where the LVEF LVEF ≥ 50%, and heart failure with mildly reduced ejection fraction (HFmEF) where the LVEF ranges from 41% to 49%.^3^, ^10^ In addition, the New York Heart Association (NYHA) classification system categorizes individuals into four groups according to their level of physical activity limitations in relation to heart failure: Classes I, II, III, and IV. These classes are characterized by increasing severity of symptoms and greater physical restrictions, with Class I representing the mildest and Class IV the most severe.^11^, ^12^

The renin‐angiotensin‐aldosterone system (RAAS) plays a significant role in the pathophysiology of HF. Angiotensin II is the primary outcome of this cascade, and it exerts several systemic effects that first serve as compensatory mechanisms but subsequently exacerbate the heart failure situation.^13^, ^14^ The strategic focus on components of the renin‐angiotensin‐aldosterone system (RAAS) has yielded significant decreases in both morbidity and mortality. Angiotensin converting enzyme (ACE) inhibitors are pharmacological agents that inhibit the enzymatic conversion of angiotensin I to angiotensin II. On the other hand, angiotensin receptor blockers (ARBs) are drugs that specifically block the angiotensin II receptors, known as AT1 receptors, which are present in the heart, blood vessels, and kidneys. This blockade leads to the dilation of blood vessels and an enhancement in blood flow.^15^, ^16^ The activation of the natriuretic peptide system (NPS), which operates in opposition to the renin‐angiotensin‐aldosterone system (RAAS) and has favorable effects on the pathophysiology of HF, also occurs during episodes of HF, resulting in elevated levels of brain natriuretic peptide (BNP) and NT‐proBNP. The NPS pathway elicits vasodilation, natriuresis, decreased blood pressure, and reduced sympathetic tone, concurrently leading to a decrease in aldosterone levels. The degradation of natriuretic peptides by neprilysin renders a pharmacological inhibitor of this enzyme beneficial.^17^, ^18^

Sacubitril‐valsartan represents a novel class of medication known as angiotensin receptor neprilysin inhibitors (ARNIs), which can serve as a viable alternative to angiotensin‐converting enzyme (ACE) inhibitors or angiotensin receptor blockers (ARBs). In the PARADIGM‐HF research,^19^ it was shown that individuals diagnosed with LVEF < 40% (HFrEF) and New York Heart Association (NYHA) class II‐IV who received sacubitril‐valsartan exhibited significant decreases in all‐cause mortality, cardiovascular mortality, and initial hospitalization due to heart failure as compared to those treated with enalapril. This led to the initiation of several more trials that aimed to compare the efficacy and safety of sacubitril‐valsartan with that of ACE inhibitors and ARBs. Recent clinical trials have also shown that sacubitril/valsartan improves left ventricular systolic and diastolic function in patients with HFrEF and end‐stage kidney disease indicating its efficacy in patients with advanced stage kidney disease that have the highest likelihood for heart failure.^20^ Furthermore, analysis of PARAGON‐HF and PARAGLIDE‐HF trials that included patients hospitalized for HF showed a significant reduction in occurrence of cardiovascular and renal events when EF > 40%.^21^

This study presents a thorough and up‐to‐date meta‐analysis of clinical studies aimed at evaluating the safety and effectiveness of sacubitril‐valsartan in comparison to RAAS inhibitors (ACE inhibitors or ARBs) as standalone treatments for patients diagnosed with heart failure.

## 2 METHODS

### 2.1 Search strategy

In November 2021, a comprehensive literature search was performed on MEDLINE (PubMed), Scopus, Embase, Web of Science, and the Cochrane Register of Controlled Trials (CENTRAL). The researcher employed a variety of search phrases, including sacubitril‐valsartan, LCZ696, ARNI, valsartan, angiotensin converting enzyme (ACE) inhibitors, HF, and angiotensin receptor blockers (ARBs), in different combinations. Furthermore, a thorough compilation of search phrases, which encompassed Medical Subject Headings (MeSH) terms, was employed. The titles and abstracts of research deemed possibly relevant were thoroughly reviewed, and afterwards, the complete text versions of the relevant papers were carefully examined. Additional papers were identified through the process of cross‐referencing the reference lists of the pertinent research.

The author conducted a thorough examination of pertinent sources to identify studies that were directly relevant to the topic at hand. The primary focus of data collection for this study involved obtaining whole papers from various sources, while abstracts were utilized to gather adequate information for the subsequent meta‐analysis. In accordance with the established inclusion criteria, obsolete references were omitted while valuable studies were incorporated. The researcher independently collected event data that included relevant variables.

### 2.2 Study selection or inclusion/exclusion criteria

Randomized controlled studies (RCT) that compared sacubitril‐valsartan were included in patients with HF with reduced ejection fraction (HFrEF, LVEF ≤ 40%), those with preserved ejection fraction (HFpEF,—LVEF ≥ 50%), and those with mildly preserved ejection fraction (HFmrEF, LVEF 41%–49%). The comparator treatment in the studies was ACE inhibitors or ARBs. All studies reporting mortality (all‐cause or cardiovascular), HF hospitalization events, and adverse events (hypotension, hyperkalaemia, worsening renal function, and angioedema) were included. Exclusion criteria were nonrandomized studies, studies with placebo comparators, with participants without heart failure, and those published in languages other than English.

### 2.3 Data extraction and quality assessment

After identifying the articles that fulfilled the inclusion criteria, the data was extracted using a predetermined data extraction form. The form encompassed the following elements: the author of the study, the name of the trial, the type of heart failure experienced by the participants, their New York Heart Association (NYHA) class, the drug used for comparison, mortality rates (including all‐cause mortality and cardiovascular mortality), hospitalizations due to heart failure, and any adverse events reported (such as hypotension, hyperkalemia, worsening renal function, and angioedema). The selected trials included a definition of hypotension, which encompassed either clinical hypotension or a systolic blood pressure (SBP) measurement below 90 mm Hg.

The methodological rigor of the papers included in the analysis was evaluated by employing the Cochrane Collaboration's risk of bias assessment.^22^ This instrument incorporates several criteria, namely randomization, allocation concealment, blinding, and completeness of follow‐up. The risk of bias for each item was assessed and categorized as high, low, or uncertain.

### 2.4 Quantitative data synthesis

The meta‐analysis was conducted using Review Manager (RevMan, Version 5. Copenhagen: The Nordic Cochrane Center, The Cochrane Collaboration. 2020). The risk ratio and its corresponding 95% confidence interval were computed based on the absolute participant counts in both the intervention and control groups.

The researchers performed meta‐analyses utilizing a random‐effects model, specifically the Mantel–Haenszel method. To evaluate the variability among the studies included in the analysis, the *I* ^2^ statistic was employed. The *I* ^2^ values were categorized as follows: values below 25% indicated low heterogeneity, values between 25% and 50% indicated moderate heterogeneity, and values exceeding 50% indicated high heterogeneity.^23^ Forest plots were generated, and a statistically significant result was seen with a *p* < .05. Furthermore, subgroup analyses were performed to examine the association between the type of heart failure (HFrEF, HFpEF, and HFmrEF) and the specific side effects. A funnel plot was employed to investigate the presence of publication bias, wherein the log risk ratio for each study was plotted against its corresponding standard error.

## 3 RESULTS

### 3.1 Identification of studies

Searching the database generated 1740 results, of which 1592 were vetted based on title and abstract. Irrelevant records (*n* = 1393) were eliminated, and 199 RCTs were evaluated for eligibility. However, 190 RCTs were removed for a variety of reasons, including inadequate comparator, trial design, result, and reviews. Figure S1 depicts the selection procedure.

### 3.2 Study characteristics

In total, 9 RCTs totaling 15 939 participants met the inclusion criteria (sacubitril‐valsartan intervention group: 7963 participants and Control group (ACE inhibitors or ARBs): (7976 participants). The studies included male and female participants (>18 years of age). Six trials were conducted in participants with HFrEF, two trials in patients with HFpEF and HFmrEF, and one trial in participants with HFrEF and HFmrEF. The features of the studies included in the meta‐analysis are shown in Table 1.

**Table 1:** Characteristics of trials included in the meta‐analysis.

References	Trial name	Trial design	NYHA class^a^	HF class^b^
Owen et al.^24^	AWAKE‐HF	Randomized, double‐blind	Class II–III	HFrEF
Desai et al.^25^	EVALUATE‐HF	Randomized, parallel	Class I–III	HFrEF
Piepoli et al.^26^	OUTSTEP‐HF	Randomized, double‐blind, prospective	Class II–IV	HFrEF
McMurray et al.^19^	PARADIGM‐HF	Randomized, double‐blind	Class II–IV	HFrEF
Tsutsui et al.^27^	PARALLEL‐HF	Randomized, double‐blind	Class II–IV	HFrEF
Solomon et al.^28^	PARAGON‐HF	Randomized, double‐blind	Class II–IV	HFpEF and HFmrEF
Solomon et al.^29^	PARAMOUNT	Randomized, double‐blind	Class II–III	HFpEF and HFmrEF
Velazquez et al.^30^	PIONEER‐HF	Randomized, double‐blind	Class I–IV	HFrEF
Kang et al.^31^	PRIME‐HF	Randomized, double‐blind	Class II–III	HFrEF and HFmrEF

Abbreviation: HFpEF, heart failure with preserved ejection fraction.

a NYHA, New York Heart Association.

b HF, heart failure class, HFrEF (LVEF ≤ 40%), HFmrEF (LVEF 41%–49%), and HFpEF (LVEF ≥ 50%).

### 3.3 Characteristics of intervention group

Sacubitril‐valsartan was administered orally in eight trials at a dose of 200 mg bid (sacubitril 97 mg and valsartan 103 mg). In one trial (PARALLEL‐HF) sacubitril‐valsartan was administered at a dose of 100 mg bid. Five trials used enalapril as a comparator at a dose of 10 mg bid, and the PARALLEL‐HF trial used enalapril in a dose of 5 mg bid. In the remaining three trials, valsartan was used as a comparator at a dose of 160 mg bid. The follow‐up periods of the trials ranged from approximately 3 months to 35 months. Supporting Information S4: Table 1 outlines information of the intervention and control groups.

### 3.4 Bias assessment

Figure S2 depicts the findings of the risk of bias assessment. Overall, the risk of bias was low in most areas. The risk of bias from randomization, allocation concealment, and detection bias categories, on the other hand, remained unclear. Although the funnel plot for outcome of all‐cause mortality looked symmetrical (Figure S3), the number of studies included was low (*n* = 8 trials) which is usually regarded as insufficient to ascertain publication bias.

### 3.5 Efficacy and safety outcomes

Supporting Information S4: Table 2 shows the results for mortality (all‐cause and cardiovascular mortality) and HF hospitalization for the trials and Supporting Information S4: Table 3 shows the adverse effects reported for the intervention and control groups for the trials reporting these outcomes. The most reported adverse events were hypotension and hyperkalaemia. The values in the tables represent the number of participants experiencing the event divided by the total number of participants in the group.

### 3.6 Meta‐analysis results

**Figure 1:**
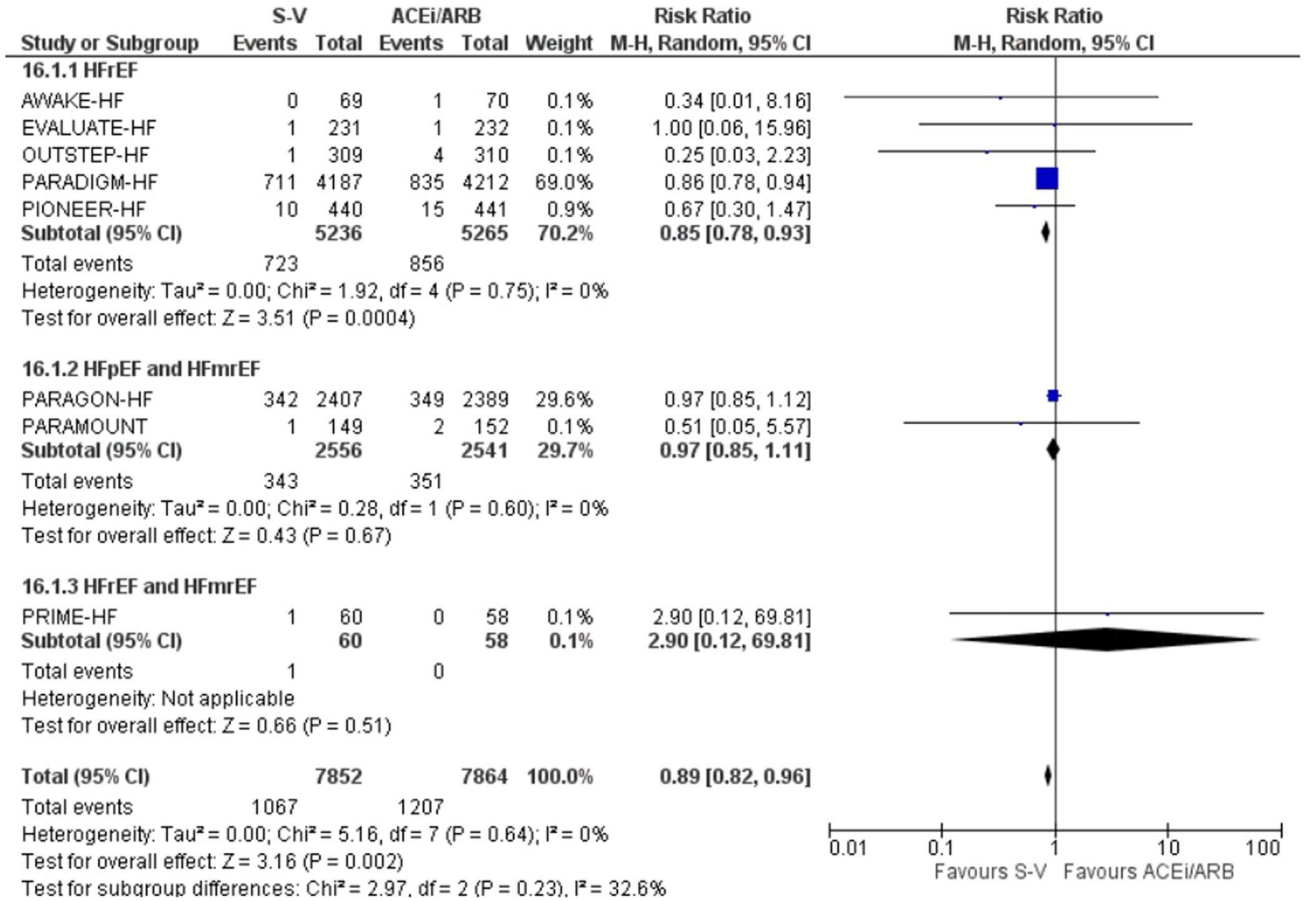
Forest plot of trials included in the meta‐analysis (*n* = 8) using a random‐effects model for all‐cause mortality outcome. Risk ratios and 95% confidence intervals are shown. ACEi/ARB, angiotensin converting enzyme inhibitors and angiotensin receptor blockers; S‐V, sacubitril‐valsartan.

In patients with HFrEF, all‐cause mortality was considerably lower in the sacubitril‐valsartan group than in the ACE inhibitors/ARBs group (RR: 0.85 [0.78, 0.93], *I* ^2^ = 0%, *p* = .0004). There was no significant difference between sacubitril‐valsartan and ACE inhibitors/ARBs for the outcome of all‐cause mortality in patients with HFpEF and HFmrEF (RR: 0.97 [0.85, 1.11], *I* ^2^ = 0%, *p* = .67). There was no significant subgroup effect (*p* = .23), indicating that the ejection fraction did not influence the efficacy of sacubitril‐valsartan (Figure 1).

**Figure 2:**
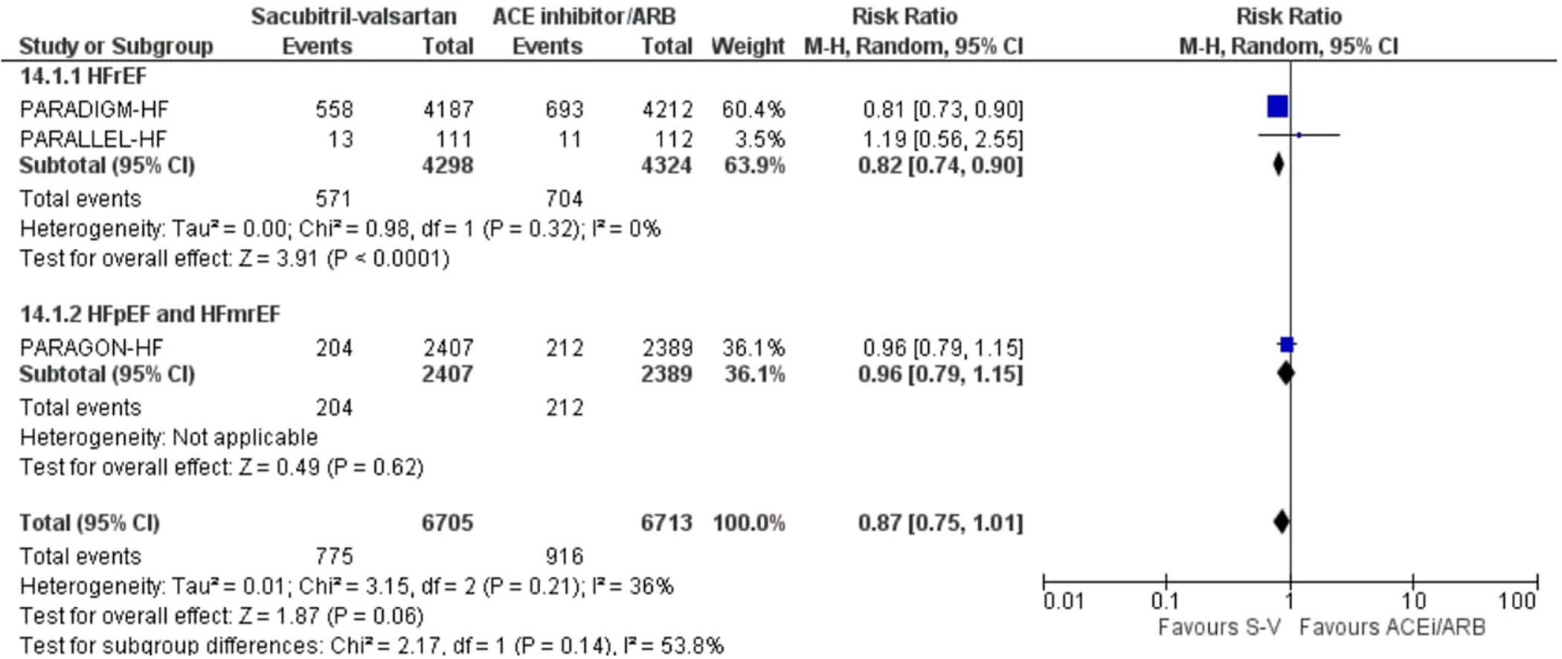
Forest plot of trials included in the meta‐analysis (*n* = 3) using a random‐effects model with cardiovascular mortality outcome. Risk ratios and 95% confidence intervals are shown. ACEi/ARB, angiotensin converting enzyme inhibitors and angiotensin receptor blockers; S‐V, sacubitril‐valsartan.

When cardiovascular mortality was used as the outcome, patients with HFrEF benefited from the use of sacubitril‐valsartan (RR: 0.82 [0.74, 0.90], *I* ^2^ = 0%, *p* < .0001) (Figure 2).

**Figure 3:**
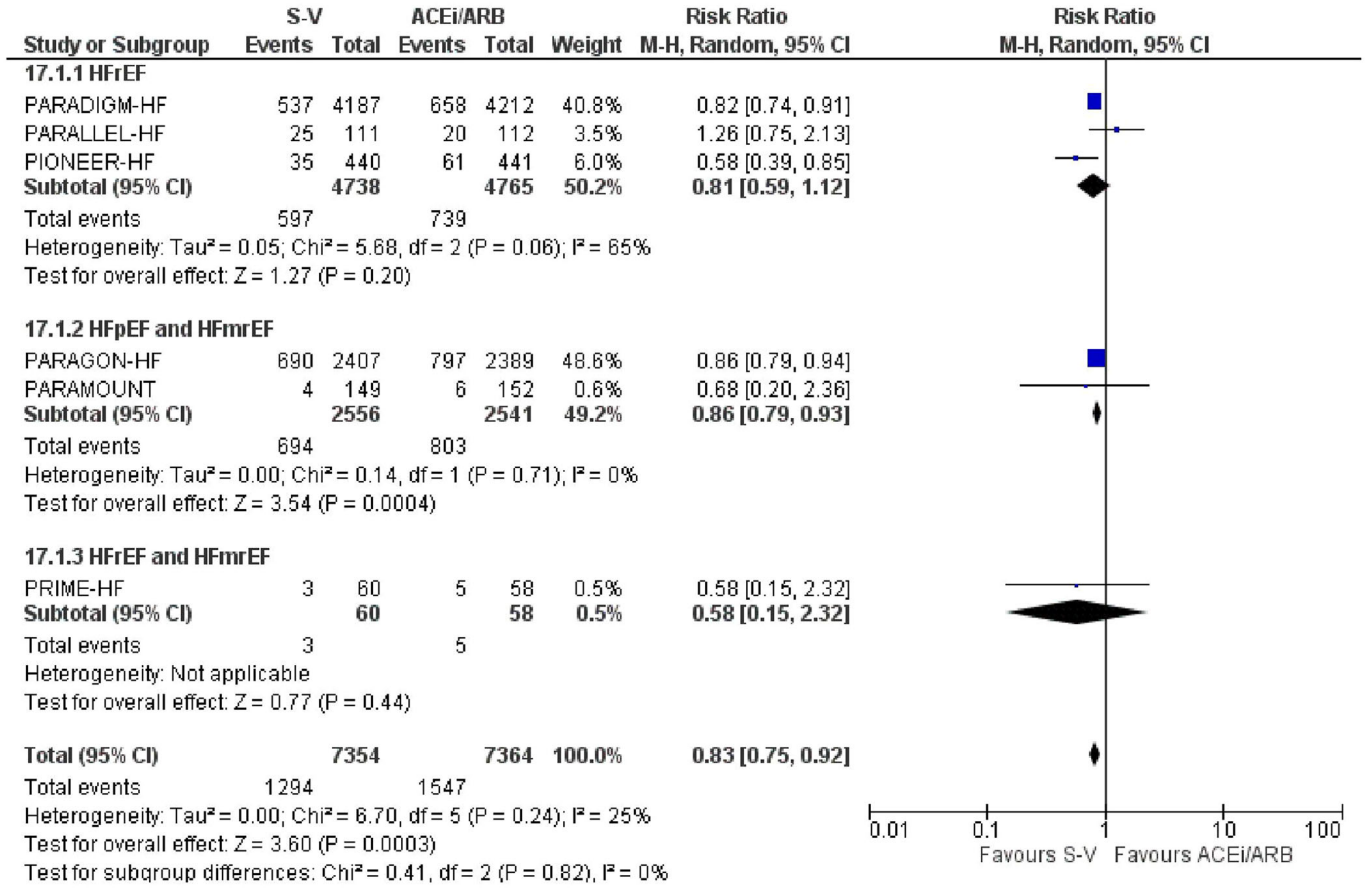
Forest plot of trials included in the meta‐analysis (*n* = 6) using a random‐effects model with HF hospitalization outcome. Risk ratios and 95% confidence intervals are shown. ACEi/ARB, angiotensin converting enzyme inhibitors and angiotensin receptor blockers; S‐V, sacubitril‐valsartan.

Sacubitril‐valsartan did not cause a decrease in hospitalization events for participants with HFrEF (RR: 0.81 [0.59, 1.12], *I* ^2^ = 65%, *p* = .20). Whereas it caused a significant decrease in hospitalizations for participants with HFpEF and HFmrEF (RR: 0.86 [0.79,0.93], *I* ^2^ = 0%, *p* = .0004). In addition, there was no significant effect of ejection fraction on hospitalization events (*p* = .82) (Figure 3).

**Figure 4:**
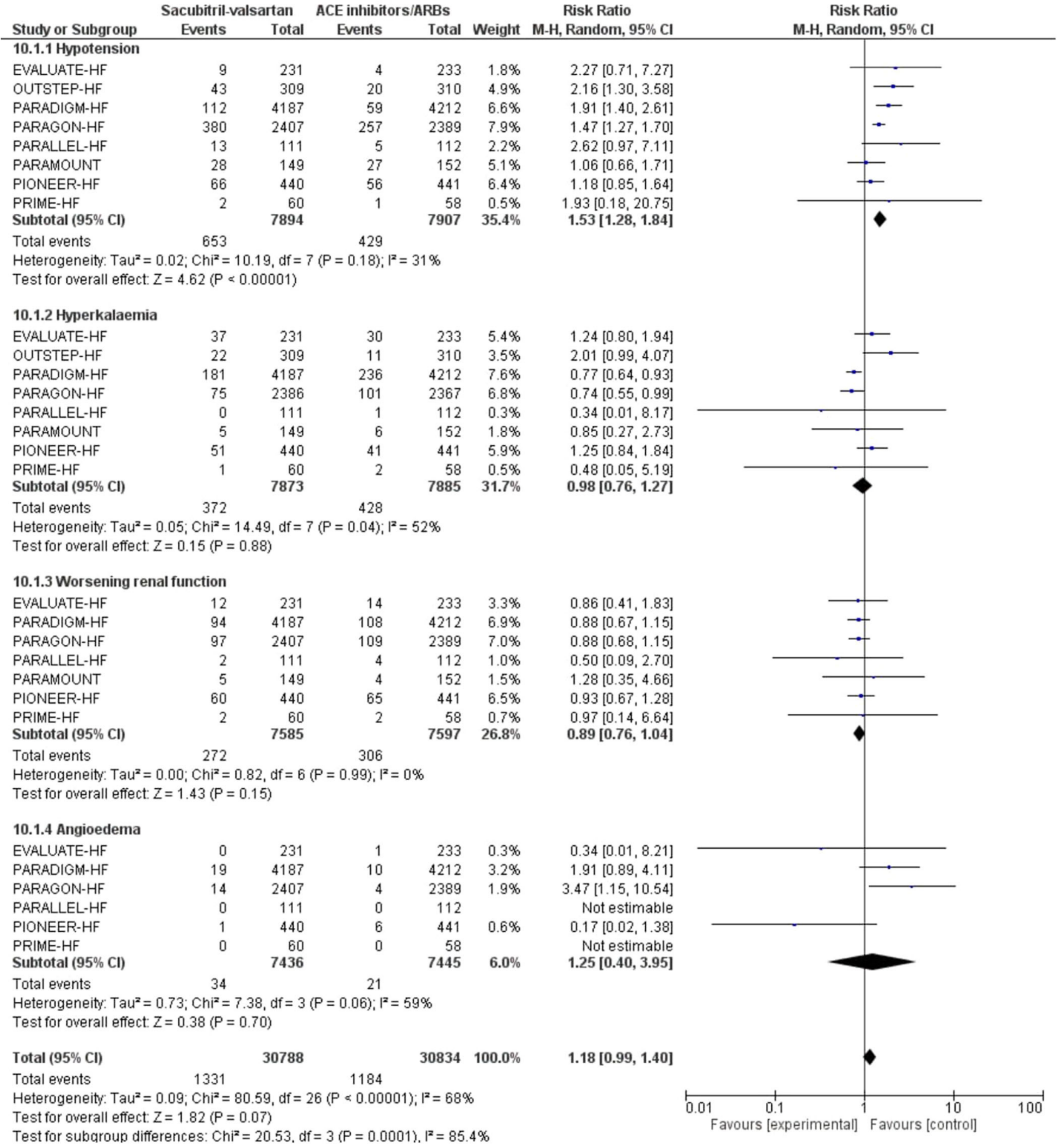
Forest plot of trials included in the meta‐analysis (*n* = 10) using a random‐effects model with adverse events outcome. Risk ratios and 95% confidence intervals are shown. ACEi/ARB, angiotensin converting enzyme inhibitors and angiotensin receptor blockers; S‐V, sacubitril‐valsartan.

Adverse events such as hypotension were significantly higher in the sacubitril‐valsartan group compared to ACE inhibitors or ARBs (RR: 1.53 [1.28, 1.84], *I* ^2^ = 31%, *p* < .00001). Other adverse events such as hyperkalaemia, worsening of renal function, and angioedema were similar between the two groups (Figure 4).

## 4 DISCUSSION

The current study provides up‐to‐date and useful information on the safety and effectiveness of sacubitril‐valsartan against RAAS inhibitors in patients with different ejection fractions categorized according to the universal classification for HF.^10^ The studies included in this meta‐analysis are all multicentre, randomized, double‐blind trials with an active comparator group with a low to moderate risk of bias across most domains. This study provides comprehensive evidence on the primary efficacy outcomes (mortality and hospitalization events) and adverse effects of sacubitril‐valsartan versus ACE inhibitors/ARBs.

In patients with HFrEF, sacubitril‐valsartan showed significant advantages in terms of lowering all‐cause and cardiovascular mortality. In individuals with HFpEF or HFmrEF, however, there was no significant impact. Among the studies in patients with HFrEF, only results from the PARADIGM‐HF study showed a reduction in all‐cause mortality when sacubitril‐valsartan was used compared to enalapril. This might be due to the PARADIGM‐HF trial's 27‐month follow‐up term, which is longer than the other trials' follow‐up periods, which range from 2 to 12 months. Solomon et al.^32^ utilized data from two studies with varied patient criteria in terms of LVEF, namely the PARADIGM‐HF trial (LVEF eligibility ≤40%) and PARAGON‐HF (LVEF eligibility ≥45%), to find a substantial reduction in all‐cause mortality and cardiovascular mortality in the HFrEF groups. This study evaluated data and outcomes using different categories of ejection fraction and showed that patients with ejection fraction lower than normal (mid‐range, borderline, or mildly reduced ejection fraction) would be expected to benefit from sacubitril‐valsartan versus RAAS inhibition. Diminished responses to sacubitril‐valsartan among patients with LVEF ≥ 50% and LVEF 41–49%%, may be related to factors such pathological processes in patients with higher LVEF range and possible amyloid deposition.^32^ However, there were only two trials for patients with HFpEF and HFmrEF indicating that the meta‐analysis results are not reliable indicators of efficacy in this LVEF category. The results of the meta‐analysis are reliable for the HFrEF category as the number of studies is sufficient and heterogeneity values low (*I* ^2^ = 0%) indicating the uniform participant and trial characteristics inspite of one of the trials that used valsartan as the comparator group (PRIME‐HF).

HF hospitalization events were not significantly different between the sacubitril‐valsartan and RAAS inhibitor groups for patients with HFrEF. However, there was a significant decrease in hospitalization events for patients with HFpEF and HFmrEF who received sacubitril‐valsartan treatment. The two studies, PARADIGM‐HF and PIONEER‐HF that showed a significant reduction in hospitalizations for patients receiving sacubitril‐valsartan both used enalapril (10 mg bid) as the comparator group. With regard to the other studies, the dose of sacubitril‐valsartan and enalapril was lower in the PARALLEL‐HF trial and valsartan was used as the comparator in PRIME‐HF trial which could have led to insignificant differences in hospitalization events. Contrary to the effects seen in HFrEF patients, patients with HFpEF and HFmrEF benefited from the use of sacubitril‐valsartan.

Sacubitril‐valsartan was shown to have a considerably greater rate of hypotension (either symptomatic or SBP < 90 mm Hg) than RAAS inhibitors. This is in line with a meta‐analysis of the effects of sacubitril‐valsartan against ARBs, which found that sacubitril‐valsartan reduced systolic and diastolic blood pressures significantly.^33^ The moderate heterogeneity seen with subgroup analysis of hypotension could be attributed to different comparators, follow‐up times, and LVEF of participants in the different trials. The higher incidence of hypotension in the sacubitril‐valsartan group can be attributed to the natriuretic potential of sacubitril which is an ARNi. Dose adjustment of sacubitril‐valsartan, blood pressure monitoring, and adjustment of concomitant diuretic use may help reduce hypotension incidence. Other adverse events such as hyperkalaemia, worsening renal function, and angioedema were not significantly different between sacubitril‐valsartan and comparator groups. In case of hyperkalaemia, two large trials, PARADIGM‐HF and PARAGON‐HF showed a significantly lower incidence of hyperkalaemia in the sacubitril‐valsartan group. Although, the comparators and the HF class was different between these trials, the follow‐up time in both was relatively longer. In addition, heterogeneity values for all analysis of adverse events outcomes were moderate to high as the trials different in participant characteristics and comparators. Overall, there was no significant difference between the groups in terms of adverse event rates.

Although this meta‐analysis provides insight into the efficacy and safety of sacubitril‐valsartan in HF patients, there are some limitations. Most of trials were sponsored by a drug company which adds a potential source of bias in showing greater efficacy of the intervention. The numbers of studies were low, and all outcomes were not reported for each trial thus limiting the data and making it difficult to assess publication bias. It is recommended that at least 10 studies with results be included in a Funnel plot as the power of the test decreases with fewer studies.^34^ Factors such as gender, age, HF type, baseline blood pressure, comparator, and follow‐up time varied between the trials which may have potentially confounded the results. In some trials, the follow‐up time was probably inadequate to capture long‐term adverse events. Several clinical trials to assess the efficacy of sacubitril‐valsartan are still ongoing and availability of these results will further strengthen and—add evidence in favor of sacubitril‐valsartan usage. Furthermore, as new studies have shown the benefit of sacubitril‐valsartan in patients with kidney failure and those with EF > 40%, additional studies in patients with these conditions will help strengthen evidence‐based medicine for use of this combination treatment compared to other commonly prescribed cardiovascular drugs.^20^

## 5 CONCLUSION

In individuals with HFrEF, sacubitril‐valsartan had a better impact than ACE inhibitors or ARBs in lowering all‐cause and cardiovascular mortality. Even though there was no substantial improvement in patients with HFpEF and HFmrEF, the number of trials was insufficient to reach a definite conclusion. There was no substantial difference in the occurrences of additional adverse events between sacubitril‐valsartan and RAAS inhibitors, other than hypotension. Additional data from ongoing clinical trials is expected to provide more information and provide a comprehensive summary on the benefits of sacubitril‐valsartan.

## AUTHOR CONTRIBUTIONS

Qing Ji developed the concept and designed the study and analyzed data, proofreading and final editing along with guarantor of the manuscript.

## CONFLICT OF INTEREST STATEMENT

The authors declare no conflict of interest.

## Supporting information

Supporting information.

Supporting information.

Supporting information.

Supporting information.

## Data Availability

Upon reasonable request, the corresponding author will provide access to the requested information.
