# Supplementary figures and images for: A meta‐analysis investigating the efficacy and adverse events linked to sacubitril‐valsartan in various heart failure subtypes
Source: Clin Cardiol. 2023 Nov 27;47(2):e24192. doi: 10.1002/clc.24192 (PMC10823544; doi:10.1002/clc.24192)

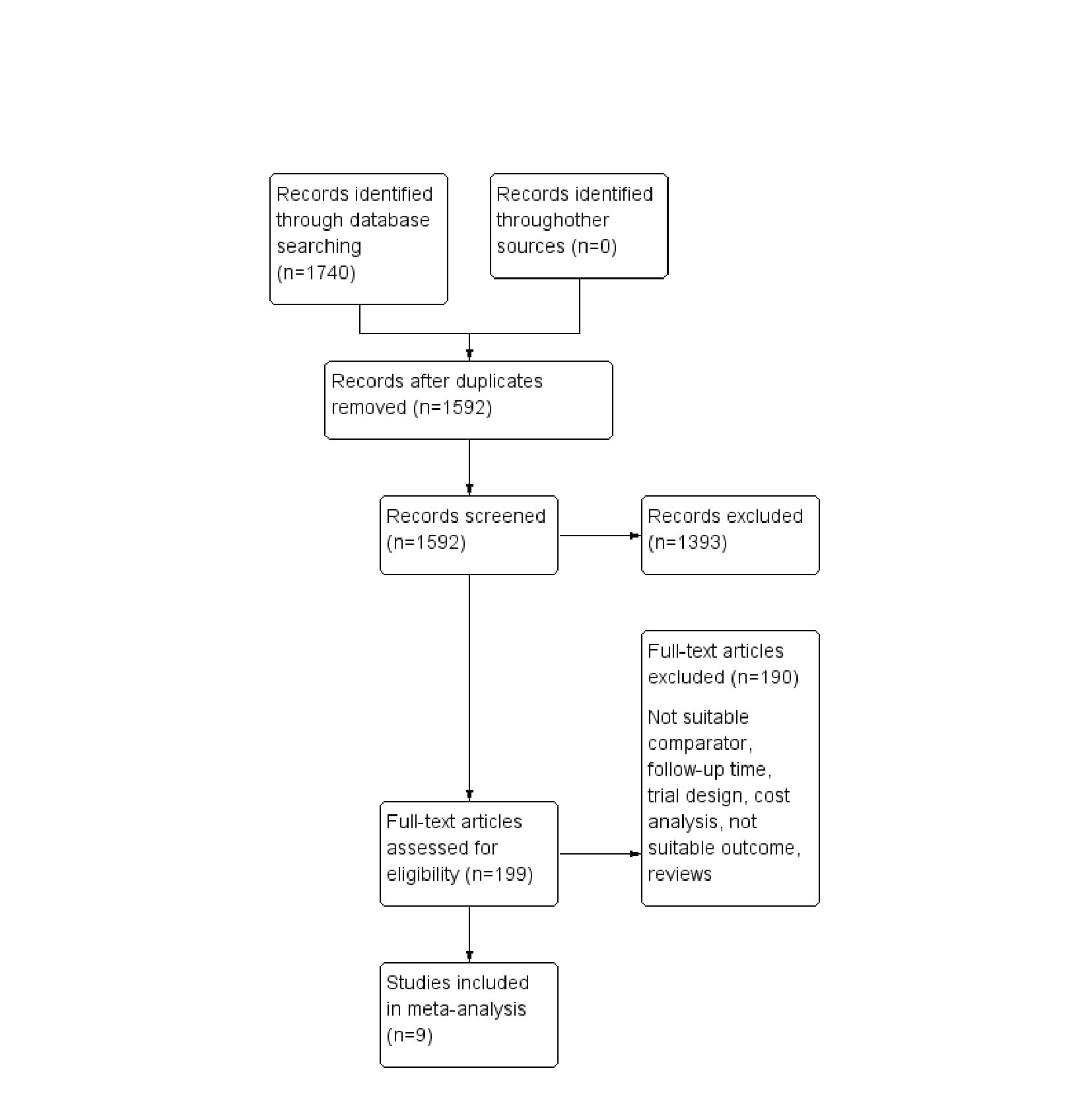

Supplement: Supplementary file 1 — Supporting information. [file CLC-47-e24192-s002.jpg]

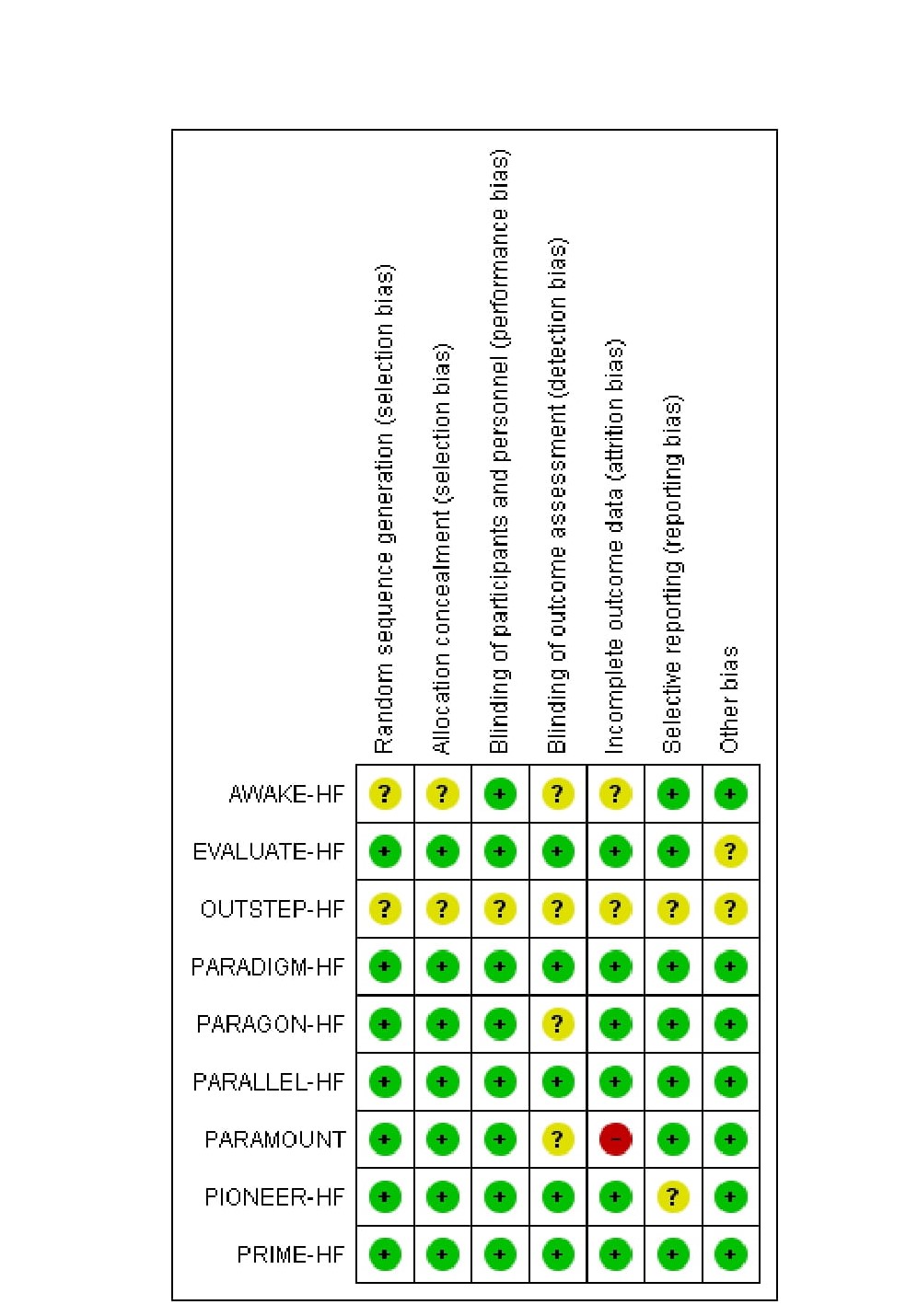

Supplement: Supplementary file 2 — Supporting information. [file CLC-47-e24192-s004.jpg]

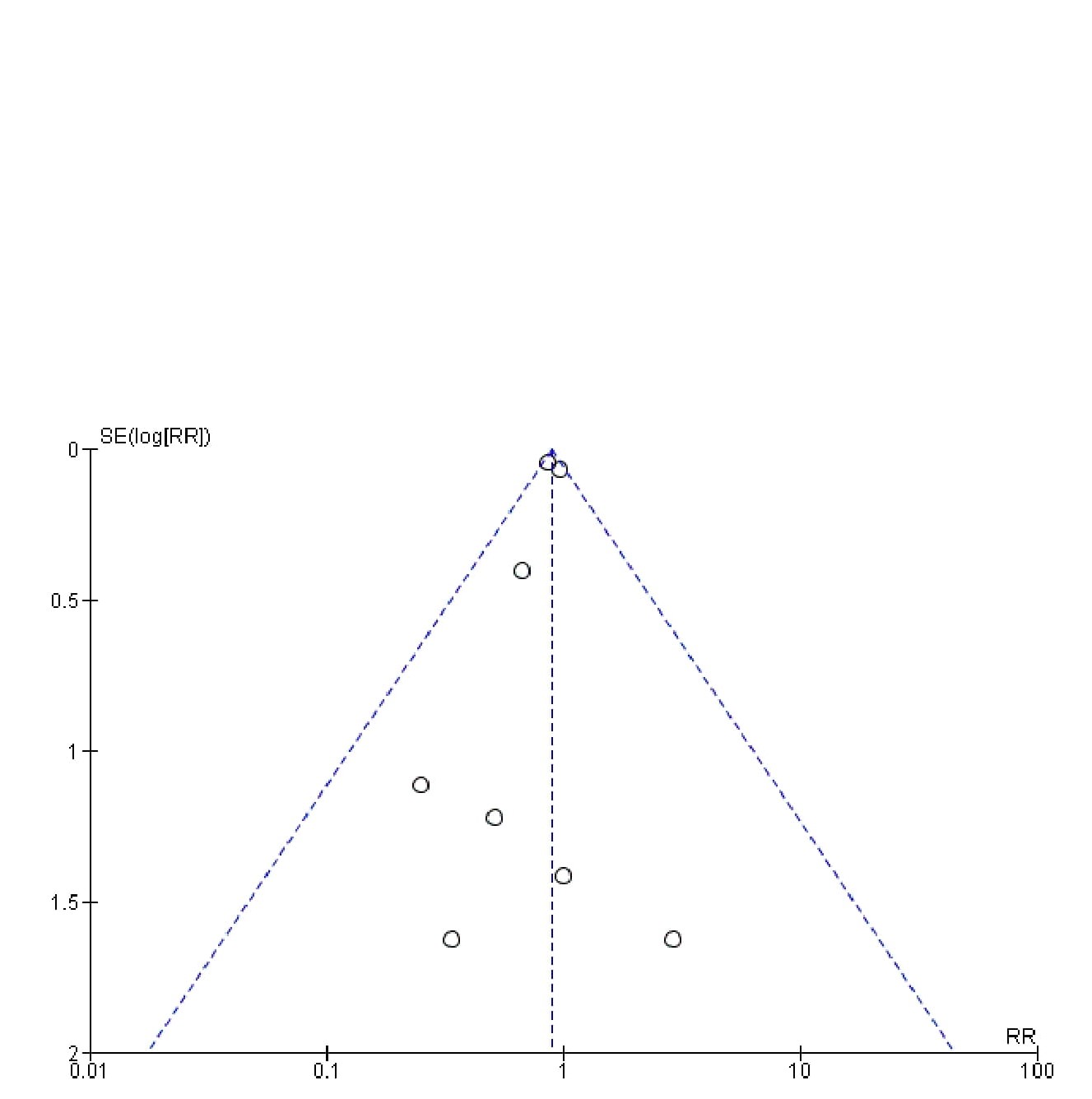

Supplement: Supplementary file 3 — Supporting information. [file CLC-47-e24192-s003.jpg]
