# Supplementary material for: A meta‐analysis investigating the efficacy and adverse events linked to sacubitril‐valsartan in various heart failure subtypes
Source: Clin Cardiol. 2023 Nov 27;47(2):e24192. doi: 10.1002/clc.24192 (PMC10823544; doi:10.1002/clc.24192)
Supplement: Supplementary file 4 — Supporting information. [file CLC-47-e24192-s001.docx]

Supplementary Table 1. Characteristics of interventions included in trials

| **Trial name** | **Intervention** | **Comparator or Control** | **Follow-up time** |
| --- | --- | --- | --- |
| AWAKE-HF (22) | Sacubitril-valsartan (200 mg bid) | Enalapril (10 mg bid) | 4 months |
| EVALUATE-HF (23) | Sacubitril-valsartan (200 mg bid) | Enalapril (10 mg bid) | 2.75 months |
| OUTSTEP-HF (24) | Sacubitril-valsartan (200 mg bid) | Enalapril (10 mg bid) | 4 months |
| PARADIGM-HF (19) | Sacubitril-valsartan (200 mg bid) | Enalapril (10 mg bid) | 27 months |
| PARALLEL-HF (25) | Sacubitril-valsartan (100 mg bid) | Enalapril (5 mg bid) | 6 months |
| PARAGON-HF (26) | Sacubitril-valsartan (200 mg bid) | Valsartan (160 mg bid) | 35 months |
| PARAMOUNT (27) | Sacubitril-valsartan (200 mg bid) | Valsartan (160 mg bid) | 21 months |
| PIONEER-HF (28) | Sacubitril-valsartan (200 mg bid) | Enalapril (10 mg bid) | 2 months |
| PRIME-HF (29) | Sacubitril-valsartan (200 mg bid) | Valsartan (160 mg bid) | 12 months |

Supplementary Table 2. Outcome data for mortality and hospitalizations of the included trials

| **Trial name** | **All-cause mortality** | **Cardiovascular mortality** | **Hospitalizations** |
| --- | --- | --- | --- |
| AWAKE-HF (22) | S-V: 0/69  E: 1/70 | NA | NA |
| EVALUATE-HF (23) | S-V: 1/231  E: 1/232 | NA | NA |
| OUTSTEP-HF (24) | S-V: 1/309  E: 4/310 | NA | NA |
| PARADIGM-HF (19) | S-V: 711/4187  E: 835/4212 | S-V: 558/4187  E: 693/4212 | S-V: 537/4187  E: 658/4212 |
| PARALLEL-HF (25) | NA | S-V: 2/111  E: 4/112 | S-V: 25/111  E: 20/112 |
| PARAGON-HF (26) | S-V: 342/2407  V: 349/2389 | S-V: 204/2407  V: 212/2389 | S-V: 690/2407  V: 797/2389 |
| PARAMOUNT (27) | S-V: 1/149  V: 2/152 | NA | S-V: 4/149  V: 6/152 |
| PIONEER-HF (28) | S-V: 10/440  E: 15/441 | NA | S-V: 35/440  E: 61/441 |
| PRIME-HF (29) | S-V: 1/60  V: 0/58 | NA | S-V: 3/60  V: 5/58 |

S-V: Sacubitril-valsartan

E: Enalapril

V: Valsartan

Supplementary Table 3. Outcomes for adverse events of the included trials

| **Trial name** | **Hypotension*** | **Hyperkalaemia**** | **Worsening renal function***** | **Angioedema** |
| --- | --- | --- | --- | --- |
| AWAKE-HF (22) | NA | NA | NA | NA |
| EVALUATE-HF (23) | S-V: 9/231  E: 4/233 | S-V: 37/231  E: 30/233 | S-V: 12/231  E: 14/233 | S-V: 0/231  E: 1/233 |
| OUTSTEP-HF (234) | S-V: 43/309  E: 20/310 | S-V: 22/309  E: 11/310 | NA | NA |
| PARADIGM-HF (19) | S-V: 2/4187  E: 59/4212 | S-V: 181/4187  E: 236/4212 | S-V: 94/4187  E: 108/4212 | S-V: 19/4187  E: 10/4212 |
| PARALLEL-HF (25) | S-V: 13/111  E: 5/112 | S-V: 0/111  E: 1/112 | S-V: 2/111  E: 4/112 | S-V: 0/111  E: 0/112 |
| PARAGON-HF (26) | S-V: 380/2407  V: 257/2389 | S-V: 75/2386  V: 101/2367 | S-V: 97/2407  V: 109/2389 | S-V: 14/2407  V: 4/2389 |
| PARAMOUNT (27) | S-V: 28/149  V: 27/152 | S-V: 5/149  V: 6/152 | S-V: 5/149  V: 4/152 | NA |
| PIONEER-HF (28) | S-V: 66/440  E: 56/441 | S-V: 51/440  V: 41/441 | S-V: 60/440  V: 65/441 | S-V: 1/440  V: 6/441 |
| PRIME-HF (29) | S-V: 2/60  V: 1/58 | S-V: 1/60  V: 2/58 | S-V: 2/60  V: 2/58 | S-V: 0/60  V: 0/58 |

S-V: Sacubitril-valsartan

E: Enalapril

V: Valsartan

*Hypotension was defined as systolic blood pressure (SBP) < 90 mm Hg or symptomatic hypotension

**Hyperkalaemia was defined as serum potassium levels ≥ 6 mmol/L

***Worsening renal function was defined as eGFR≥35% or an increase in serum creatinine ≥0.5 mg/dl from the baseline value
